# An Incidental Finding of a Large Pericardial Cyst

**DOI:** 10.7759/cureus.23917

**Published:** 2022-04-07

**Authors:** Younus Qamar, Maryam Gulzar, Amna Qamar, Haytham Sabry, Tariq Minhas

**Affiliations:** 1 Department of Cardiothoracic Surgery, The Essex Cardiothoracic Centre, Basildon & Thurrock University Hospital, Mid and South Essex NHS Foundation Trust, Basildon, GBR; 2 Department of Cardiothoracic Surgery, School of Medicine, University of Liverpool, Liverpool, GBR; 3 Department of Cardiothoracic Surgery, Liverpool Heart and Chest Hospital NHS Foundation Trust, Liverpool, GBR

**Keywords:** incidental radiological finding, congenital heart disease, video-assisted thoracoscopic surgery, mediastinal cyst, mediastinal masses, mediastinal neoplasms, mediastinal tumors, pericardial cyst, inflamed pericardial cyst, congenital pericardial defect

## Abstract

A pericardial cyst is a rare and benign cause of a mediastinal mass. They are frequently asymptomatic and are usually incidental findings on imaging. Symptoms may include persistent cough, atypical chest pain, dysphagia, and dyspnea. Diagnosis is usually established with the aid of imaging, including a chest x-ray, a computed tomography (CT) scan, and magnetic resonance imaging (MRI). Therapeutic options include surgical resection or aspiration for large and/or symptomatic cysts, whereas conservative management with routine follow-up is advised for small or asymptomatic cysts. We herein describe the case of a 48-year-old lady, who presented with clinical features suggestive of acute cholecystitis, with an incidental finding of a pericardial cyst, measuring approximately 10.1 cm x 8.7 cm x 10.7 cm. The patient underwent video-assisted thoracoscopic surgery (VATS) for excision of the pericardial cyst. She had an uneventful recovery and was discharged on the second post-operative day. At six months, there was no evidence of disease recurrence.

## Introduction

Pericardial cysts are rare and benign lesions typically located in the anterior or middle mediastinum. They account for approximately 33% of all mediastinal cysts and 6% of all mediastinal masses [1]. They are frequently asymptomatic and usually diagnosed incidentally on imaging. The peak incidence is between the age of 30 and 50 years with no gender predilection [2,3]. Occasionally, they may present with atypical chest pain, persistent cough, worsening dyspnea, and dysphagia [4,5]. Congenital pericardial cysts are thought to occur due to an aberrancy in the formation of coelomic cavities. However, they can be a sequela of pericarditis or other inflammatory/infectious processes (e.g., rheumatic fever, tuberculosis) [5]. Histologically, the wall of the cyst is composed of fibrocollagenous connective tissue and a single layer of mesothelial cells, and usually contains serous fluid [6]. Anatomically, pericardial cysts are commonly located within the right hemithorax, particularly at the right cardiophrenic angle [7]. A common variant of a pericardial cyst is called a migrating or “wandering” pericardial cyst, which describes a pedunculated pericardial cyst that may alter its position on serial imaging [6]. Pericardial cysts appear as a water-density, soft tissue mass, usually at the cardiophrenic angle on a chest x-ray [4]. Most pericardial cysts are benign in nature, and hence, a conservative approach with routine follow-up is adopted for small, asymptomatic pericardial cysts. Conversely, for symptomatic or rapidly enlarging pericardial cysts, the treatment of choice is surgical excision or percutaneous aspiration [4]. We report a case of a 48-year-old lady, who had an incidental finding of a pericardial cyst on a routine chest x-ray performed to investigate an unrelated disease.

## Case presentation

**Figure 1:**
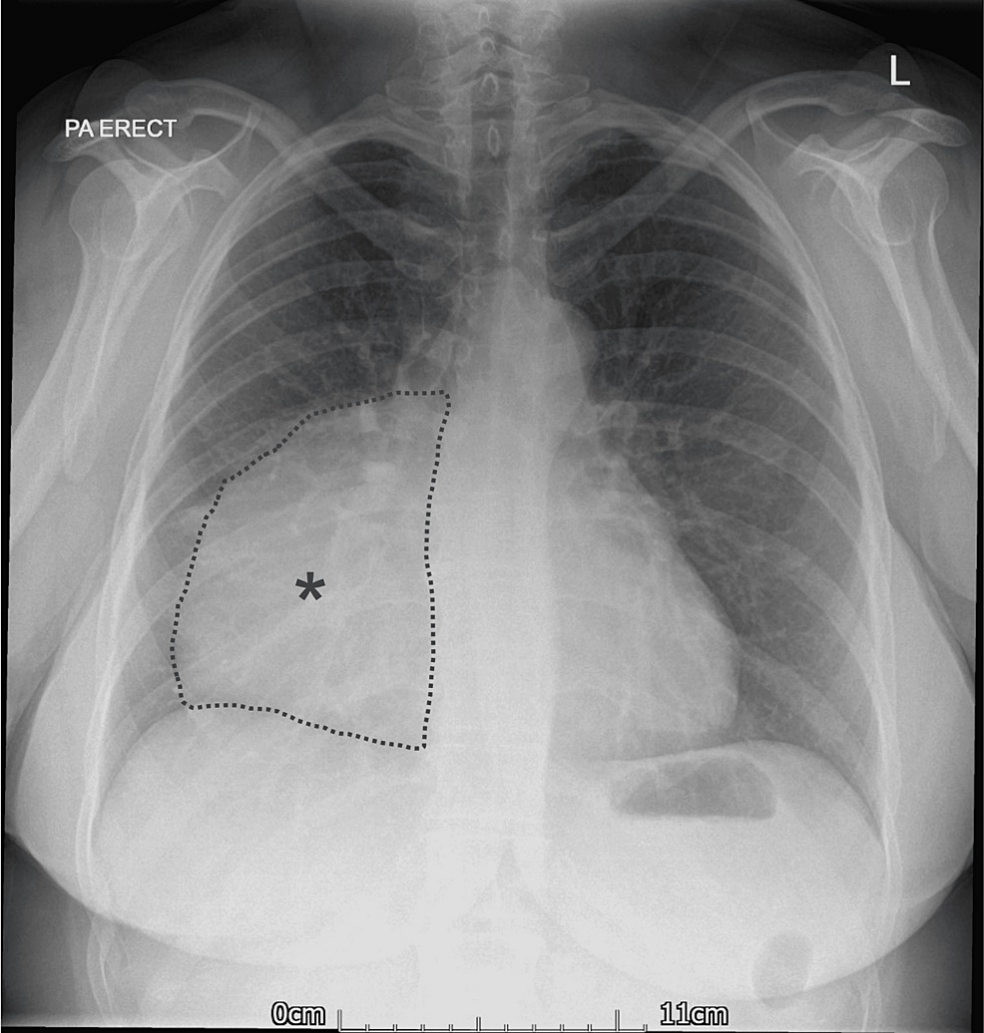
Posteroanterior (PA) chest x-ray showing a soft tissue-density consolidation in the right mid-and-lower hemithorax with obliteration of the right cardiac border (*).

A 48-year-old Caucasian lady presented to our emergency department with a three-day history of colicky abdominal pain. She had a past medical history of hypertension and asbestos exposure. She was an ex-smoker with a 40-pack-year history. At the time of admission, she was afebrile, and her vital signs were within normal limits. Clinical examination revealed mild right upper quadrant (RUQ) tenderness with a negative Murphy's sign. Blood investigations were unremarkable with a C-reactive protein (CRP) of 1 mg/mL and a white-cell count (WCC) of 6.5 x 10^9^/L. A 12-lead electrocardiogram (ECG) showed normal sinus rhythm. A plain chest x-ray showed a soft tissue mass in the right mid-and-lower hemithorax with obliteration of the heart border (Figure 1). The left lung field appeared clear with no evidence of a pleural effusion. A transthoracic echocardiogram (TTE) revealed normal left ventricular (LV) function, no regional wall motion abnormalities, and a cystic lesion near the right atrium. There was no valvular heart disease and no sonographic evidence of pericardial effusion.

**Figure 2:**
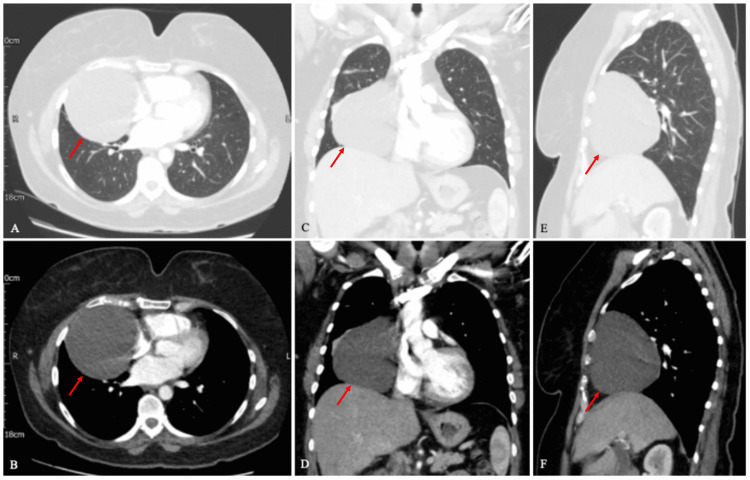
A computed tomography (CT) scan of the chest showing a large, homogenous, well-circumscribed lesion in the right lower hemithorax, and abutting the right cardiac border (red arrow). (A, C, E) Lung window (demonstrating the lung parenchyma in detail, including the pulmonary vasculature) in axial, coronal, and sagittal views, respectively. (B, D, F) Mediastinal window (demonstrating the chest wall and pleura) in axial, coronal, and sagittal views, respectively.

A computed tomography (CT) scan of the chest and liver was performed to determine the nature of this soft tissue mass. CT showed a large, homogenous, well-circumscribed, water-density mass, measuring approximately 9.9 cm x 8.8 cm x 11.5 cm, sitting at the right lower hemithorax, and abutting the right cardiac border (Figures 2A-2F). There was no evidence of surrounding lobar collapse or consolidation. The hilar lymph nodes appeared normal in size and there were no signs of mass effect. There was mild atelectasis at the superior border of the right middle lobe. Otherwise, the remaining lung parenchyma appeared unremarkable bilaterally. Given the density of the mass, it was suggested that the appearance was in keeping with a pericardial cyst. Additionally, as expected, the gallbladder was distended and appeared thick-walled, which was consistent with the diagnosis of acute cholecystitis. Moreover, there was evidence of minimal fat stranding around the gallbladder. The patient was initially managed conservatively for the acute cholecystitis with a course of intravenous antibiotics followed by a laparoscopic cholecystectomy several weeks later.

**Figure 3:**
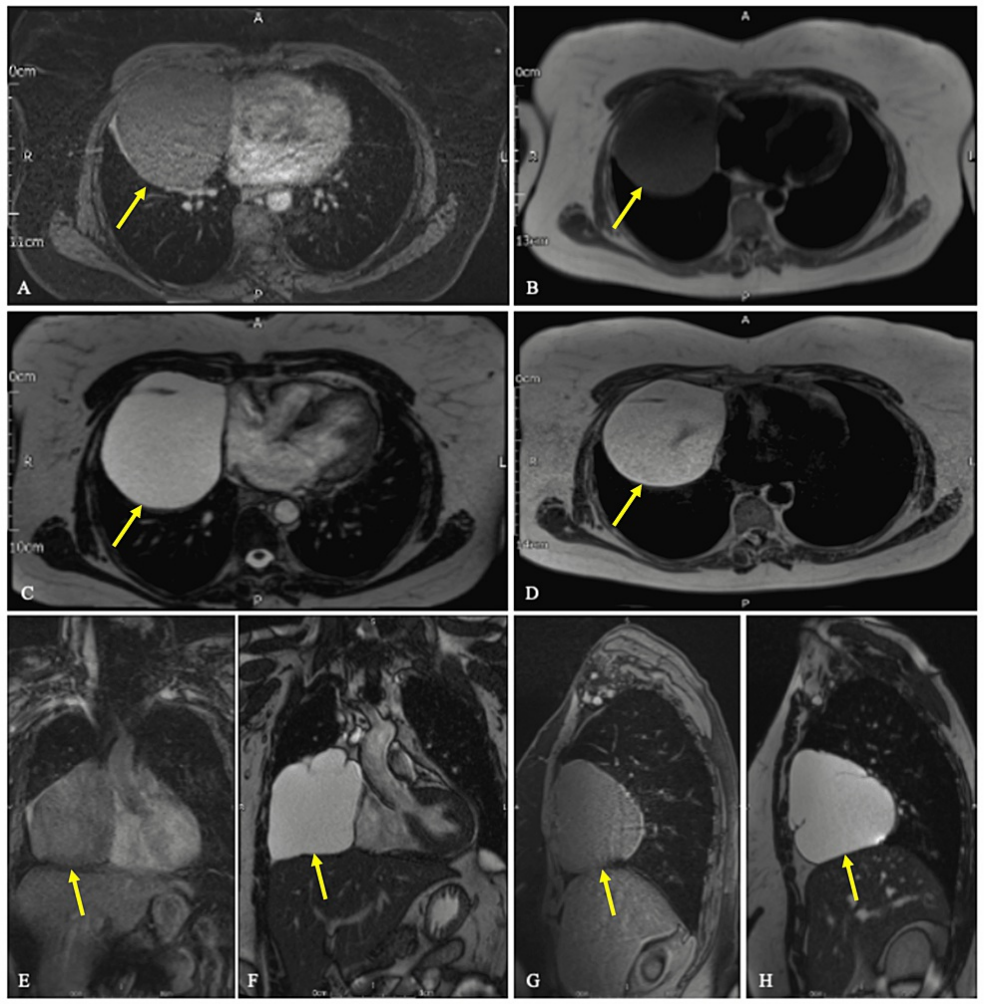
A magnetic resonance imaging (MRI) scan of the chest with gadolinium enhancement. Axial T1-weighted fat-saturated (A) and T1-weighted (B) sequences at the same level showing a water-density lesion with thin walls, located at the cardiophrenic angle (yellow arrow). (C, D) T2-weighted and half-Fourier acquired single-shot turbo-spin echo (HASTE) sequences in axial view at the same level, respectively. (E, F) Coronal T1-weighted and T2-true fast imaging with steady-state-free precession (TRUFI) sequences, respectively. Sagittal T1-weighted (G) and T2-TRUFI (H) sequences.

This study was supplemented with a magnetic resonance imaging (MRI) scan of the chest with gadolinium enhancement. MRI showed a cystic lesion abutting the right side of the heart, isointense to the myocardium, on T1-weighted sequences. The cystic lesion was hyperintense on T2-weighted sequences. It measured approximately 10.1 cm x 8.7 cm x 10.7 cm. The cyst was thin-walled with two thin septations, and no solid component (Figures 3A-3H). There were no areas of contrast enhancement and no associated pericardial effusion. Furthermore, there were no enlarged mediastinal lymph nodes. The location and morphology of the cystic lesion were consistent with a benign, pericardial cyst.

**Figure 4:**
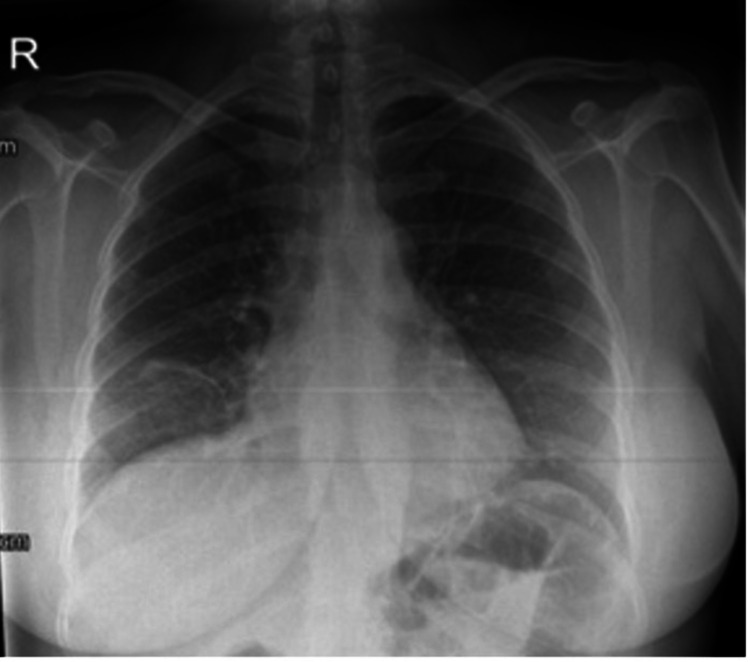
Posteroanterior (PA) chest x-ray, performed on the sixth post-operative day, demonstrating clear lung fields bilaterally, with no obvious masses, nodules, consolidation or collapse visible. The previously seen soft tissue mass is no longer present. The heart was not enlarged and the cardiomediastinal contours were normal.

The patient was reviewed by the thoracic surgeons and a decision was made to proceed with video-assisted thoracoscopic surgery (VATS) for excision of the pericardial cyst. The procedure was performed under general anesthesia with the use of a 35 French left-sided double-lumen endotracheal tube (ETT). Surgical access was obtained via a 3 cm skin incision through the fifth intercostal space (5^th ^ICS), slightly anterior to the mid-axillary line. Upon entering the chest cavity, a large cystic lesion was identified, adherent to the pericardium. There was no solid component and no obvious communication with the pericardium. The pericardial cyst was excised using the so-called "piecemeal" resection. A remnant of the pericardial cyst was left in situ, which was tethered to the right phrenic nerve. All specimens obtained were sent for histopathological analysis. The patient made an uneventful recovery post-operatively and was safely discharged from the hospital on the second post-operative day. A chest x-ray, performed prior to her discharge, showed that both lung fields were clear with no obvious masses, nodules, consolidation, or collapse visible. The heart was not enlarged and the cardiomediastinal contours were normal (Figure 4).

Histopathological analysis of the resected specimen confirmed the diagnosis of a benign, chronically inflamed, pericardial cyst. It showed a cyst wall composed of a thin layer of fibrous tissue lined by mesothelial cells with reactive features. The underlying fibroadipose tissue showed a patchy lymphoid infiltrate including scattered lymphoid aggregates with reactive germinal center formation. Immunohistochemistry for pancytokeratin (AE1/AE3) stained the mesothelial lining only. The lymphoid infiltrate contained a significant amount of CD20 positive B cells, alongside CD3 and CD5-positive T cells. Overall, the cyst was composed of a thin fibrous wall lined by mesothelial cells. Underlying this was a lymphocytic cell infiltrate composed of primary and secondary follicles. There were no phenotypically abnormal B-cells, and the rate of proliferation was low. These features were suggestive of a post-infectious etiology for the pericardial cyst. At six months, a follow-up chest x-ray demonstrated no features of disease recurrence.

## Discussion

Pericardial cysts are rare, benign, congenital causes of a mediastinal mass with an incidence of one in 100,000 [1]. They are typically located within the anterior or middle mediastinum [7]. They account for approximately 33% of all mediastinal cysts and 6% of all mediastinal masses [1]. Although they are commonly congenital, other causes of a pericardial cyst include post-infection (e.g., pericarditis, tuberculosis, rheumatic fever), post-cardiac surgery, and in the setting of autoimmune diseases [5]. They are typically located within the right hemithorax, particularly at the cardiophrenic sulcus, with approximately 22% located within the left hemithorax [7]. Congenital pericardial cysts are thought to result from an aberrancy in the formation of coelomic cavities. There is an incomplete coalescence of fetal lacunae during the formation of the pericardial sac. Histologically, these cysts are composed of a single layer of mesothelial cells in a stroma of fibrocollagenous connective tissue. They usually contain serous fluid giving them a water-like density on imaging [6]. They can vary considerably in size, ranging from 2 to 15 cm in dimension [1,4].

Pericardial cysts are usually asymptomatic and are frequently an incidental finding on a chest x-ray performed to investigate an unrelated disease. Symptoms may include atypical chest pain, worsening dyspnea, dysphagia, or persistent cough, and is due to compression from the cyst itself on the surrounding structures (i.e., the lung parenchyma and the heart). Rarely, it may cause life-threatening complications such as cyst infection, cardiac tamponade, obstruction of the right main bronchus, arrhythmias (including atrial fibrillation), right ventricular outflow tract (RVOT) obstruction, and sudden cardiac death. The peak incidence is between the age of 30 to 50 years with no gender preference [2,3].

On a chest x-ray, a pericardial cyst appears as a well-defined, soft tissue mass, usually at the right cardiophrenic angle [4]. The characteristic appearance on a CT scan is a homogenous, fluid-filled lesion, which is well-circumscribed and has thin walls. The cyst would typically have an attenuation coefficient of 0-20 Hounsfield Unit (HU) because of its serous fluid content [4,8]. MRI is the imaging modality of choice and is usually diagnostic, providing detailed anatomical description and tissue characterization. The fluid content of the pericardial cyst is reflected by homogenous signal hyperintensity on T2-weighted sequences and homogenous signal hypointensity on T1-weighted sequences. They do not typically enhance following administration of contrast (e.g., gadolinium) [1,4,8]. A TTE may be useful in evaluating for the mass effect of the cystic lesion on cardiac function. It can help determine whether there is an associated pericardial collection or effusion, and if the cyst itself communicates with the pericardium (as seen with a pericardial diverticulum). Moreover, it can help differentiate a pericardial cyst from other masses, including a prominent fat pad, ventricular aneurysm, an appendage, and solid tumors [1,5,9].

Once the diagnosis of a benign pericardial cyst has been established, the therapeutic options include surgical excision or percutaneous aspiration for large and/or symptomatic cysts. Alternatively, a small or asymptomatic cyst is best managed conservatively with routine chest x-ray or CT scans to monitor for growth in the size of the cystic lesion [4]. However, the frequency or time interval for follow-up imaging has not been established. Furthermore, there is no consensus on the appropriate time period for which such cases should be monitored. Hence, each case is managed on its own merits. In symptomatic individuals with a large pericardial cyst, the treatment of choice is surgical excision via a VATS, thoracotomy, midline sternotomy, or mediastinoscopy. A complete resection must be performed to avoid disease recurrence [1,4]. Alternatively, an ultrasound or echocardiogram-guided percutaneous aspiration of the pericardial cyst may be considered, particularly in patients who are not suitable candidates for surgery. However, we must elude them to potential risk associated with a percutaneous aspiration, including the risk of hemorrhaging into the cyst or that of disease recurrence (~30%) [4].

In this patient, the soft tissue mass identified on the chest x-ray was diagnosed as a post-inflammatory pericardial cyst, based on the histopathological and immunohistochemical findings. Other rarer causes of an acquired pericardial cysts include echinococcosis (causes a hydatid cyst to form) or trauma. This patient denied any history of chest trauma. Echinococcosis was ruled out in our patient based on her geographical location, the number and nature of the cyst itself, and the absence of echinococcosis on histopathology [10]. Pericardial cysts generally have a favorable prognosis; however, its natural history is not fully known [1-4].

## Conclusions

In the case described above, the patient was asymptomatic from a cardiopulmonary point of view, and the pericardial cyst was found incidentally on a routine chest X-ray. The diagnosis was confirmed with the aid of CT and MRI scans. Although a pericardial cyst is a rare entity, it should be considered in the differential diagnosis of mediastinal masses. Moreover, they are clinically heterogeneous; they are largely asymptomatic, however, may present with rare, but serious, complications, including obstruction of the right main bronchus/RVOT, cardiac tamponade, and sudden cardiac death. VATS is a minimally invasive procedure, which provides a curative treatment and is associated with minimal pain and a shorter post-operative recovery period.
